# A Review of Heterogeneity in Attention Deficit/Hyperactivity Disorder (ADHD)

**DOI:** 10.3389/fnhum.2019.00042

**Published:** 2019-02-11

**Authors:** Yuyang Luo, Dana Weibman, Jeffrey M. Halperin, Xiaobo Li

**Affiliations:** ^1^Department of Biomedical Engineering, New Jersey Institute of Technology, Newark, NJ, United States; ^2^Department of Psychology, Queens College of the City University of New York, Flushing, NY, United States; ^3^Department of Electric and Computer Engineering, New Jersey Institute of Technology, Newark, NJ, United States

**Keywords:** ADHD, heterogeneity, risk factors, cognitive impairments, structural MRI, functional MRI, DTI, neuroscience model

## Abstract

Attention-deficit/hyperactivity disorder (ADHD) is a neurodevelopmental disorder that affects approximately 8%–12% of children worldwide. Throughout an individual’s lifetime, ADHD can significantly increase risk for other psychiatric disorders, educational and occupational failure, accidents, criminality, social disability and addictions. No single risk factor is necessary or sufficient to cause ADHD. The multifactorial causation of ADHD is reflected in the heterogeneity of this disorder, as indicated by its diversity of psychiatric comorbidities, varied clinical profiles, patterns of neurocognitive impairment and developmental trajectories, and the wide range of structural and functional brain anomalies. Although evidence-based treatments can reduce ADHD symptoms in a substantial portion of affected individuals, there is yet no curative treatment for ADHD. A number of theoretical models of the emergence and developmental trajectories of ADHD have been proposed, aimed at providing systematic guides for clinical research and practice. We conducted a comprehensive review of the current status of research in understanding the heterogeneity of ADHD in terms of etiology, clinical profiles and trajectories, and neurobiological mechanisms. We suggest that further research focus on investigating the impact of the etiological risk factors and their interactions with developmental neural mechanisms and clinical profiles in ADHD. Such research would have heuristic value for identifying biologically homogeneous subgroups and could facilitate the development of novel and more tailored interventions that target underlying neural anomalies characteristic of more homogeneous subgroups.

## Introduction

Attention-deficit/hyperactivity disorder (ADHD) is among the most commonly diagnosed neurodevelopmental disorders, affecting approximately 8%–12% of children worldwide, with up to 65% continuing to have ADHD symptoms and neuropsychological impairments in adulthood (Faraone et al., 2003; Polanczyk et al., 2015). The symptoms of ADHD negatively impact many aspects of individuals’ lives, families, and society, including but not limited to, educational and social outcomes, strained parent-child relationships, and increased utilization of and spending on healthcare services. Previous studies have repeatedly emphasized that ADHD is a heterogeneous disorder, in terms of the multifactorial etiological risk factors, diverse expressions of the symptom domains, comorbid disorders, neuropsychological impairments, and long-term trajectories (Castellanos et al., 2006; Costa Dias et al., 2015). The etiological heterogeneity in terms of the biological and environmental factors is likely reflected in variation in neural correlates (Nigg and Casey, 2005; Mackie et al., 2007; Fair et al., 2012; Costa Dias et al., 2013, 2015; Karalunas et al., 2014), and results in the diverse cognitive and behavioral profiles and developmental trajectories of the disorder (Halperin and Schulz, 2006; Rajendran et al., 2013; Schulz et al., 2017).

Existing research suggests that genetic variants, and pre- and peri-natal risk factors relate to the manifestation of ADHD symptoms, and appear to be associated with various neurodevelopmental and psychiatric outcomes (Bonvicini et al., 2018; Uchida et al., 2018). Currently, the diagnosis of ADHD is characterized by age-inappropriate symptoms of inattention and/or hyperactivity-impulsivity, categorized into three presentations including predominantly inattentive, predominantly hyperactive/impulsive and combined presentation, based on the Diagnostic and Statistical Manual of mental disorders, fifth edition (DSM-5; American Psychiatric Association). A diagnosis of ADHD is typically determined by a clinician based on the number, severity, and duration of behavioral symptoms observed by parents/caregivers and teachers. They are not defined based on etiological sources, or any biologically identified markers. It still remains an open question about the relations between the clinical definitions, etiological sources and neurobiological substrates of ADHD. As we review below, a large body of research has attempted to clarify their links.

A wide range of comorbid behavioral and psychiatric conditions are associated with ADHD, including learning disabilities, language disorders, mood disorders, anxiety, and conduct/oppositional disorder (Reale et al., 2017). These comorbid problems can complicate both diagnosis and treatment of ADHD.

Neurocognitive impairments are hypothesized as a core part of ADHD symptomatology (Castellanos and Tannock, 2002; van Lieshout et al., 2017; Kofler et al., 2018). Neurocognitive impairments including, but not limited to, domains of sustained attention or vigilance, executive function (EF), working memory, and self-regulation, have been frequently reported in individuals with ADHD. Notably, the nature of neurocognitive deficits is highly variable across individuals and some have no such difficulties (Nigg et al., 2005; Willcutt et al., 2005). Widespread structural and functional brain abnormalities have also been linked to ADHD. Frontal lobe, thalamus and striatum, which are the key components of the cortico-striato-thalamo-cortical (CSTC) loops that subserve attention and cognitive processing, have been found to be involved in ADHD pathophysiology (Bush et al., 2005; Li et al., 2012; Xia et al., 2012; Shaw et al., 2013). Studies suggest that functional and structural deficits in frontal lobe and thalamus are associated with the emergence of ADHD (Proal et al., 2011; Xia et al., 2012; Cortese et al., 2013; Shaw et al., 2015); while developmental diminution of the ADHD symptoms may correlate with the maturation of frontal cortex and related circuits subserved by the CSTC loops (Makris et al., 2007; Halperin et al., 2008; Proal et al., 2011; Clerkin et al., 2013; Francx et al., 2015, 2016; Schulz et al., 2017). However, neuroimaging and neuropsychological studies report inconsistent results. Although reduced frontal lobe volume has been frequently reported, there are discrepancies as to whether the anomaly: (1) was unilateral (Li et al., 2007; Ambrosino et al., 2017) or bilateral (Gehricke et al., 2017); (2) involved only inferior (Stevens and Haney-Caron, 2012), only superior (Hill et al., 2003), or both areas (Ambrosino et al., 2017); (3) comprised white matter (WM; Filipek et al., 1997), gray matter (GM; Durston, 2003), or both (Makris et al., 2007; Bush, 2010); and (4) reflected overall smaller brain volume in children with ADHD (Castellanos and Proal, 2012), or not (Durston, 2003). Similarly, although multiple studies have provided support for impairment in EF believed to be mediated by prefrontal circuits (Barkley, 1997), broader investigations found that only 50%–60% of those with ADHD were impaired on the most sensitive EF measures (i.e., Stop Signal Reaction Time; Nigg et al., 2005; Willcutt et al., 2005). Beyond possible demographic and developmental factors, etiological heterogeneity of the disorder can be a significant component that contributes to the inconsistency of these findings.

This review article will examine existing studies that focused on understanding the etiology, clinical profile, trajectories, neurocognitive impairments, and neurobiological and pathological mechanisms of ADHD. We will discuss the common and inconsistent findings in each of these aspects, and the potential influence of the heterogeneity on inconsistent findings.

## Heterogeneity of Etiological Risk Factors in ADHD

Family-based studies have consistently found higher rates of ADHD in parents and siblings of affected probands, compared to the biological relatives of unaffected controls (Chen et al., 2008). Twins studies showed that monozygotic twin pairs have much higher concordance rates for ADHD than dizygotic twin pairs (Faraone et al., 2005). Adoption studies reported increased rates of ADHD in the biological parents of ADHD adoptees, compared to both the adoptive parents of the probands and parents of controls without ADHD (Sprich et al., 2000). All these studies suggest a strong genetic component of the disorder, with heritability estimates of 60%–80% (Faraone et al., 2005).

In the past three decades, molecular genetic association studies (Faraone et al., 2005; Gizer et al., 2009), linkage studies (Ogdie et al., 2004), meta-analyses (Neale et al., 2010), and recent reviews (Thapar et al., 2013; Klein et al., 2017) have identified a number of genes that might contribute to the onset of childhood ADHD (cADHD), including, but not limited to, dopamine receptor genes such as DRD4, DRD5, DRD2, DRD3, dopamine-beta-hydroxylase (DBH), dopamine transporter gene (DAT, SLC6A3), norepinephrine transporter gene (SLC6A2), noradrenergic receptor genes such as ADRA2A, 2C, and 1C, monoamine oxidase-A (MAO-A), catechol-O-methyltransferase (COMT) serotonin receptor and transporter genes including HTR2A, HTR1B, 5-HTT, SLC6A4, and at least one GABA gene, GABRB3. However, results have been inconsistent and many findings have not been consistently replicated. For example, multiple candidate-gene association studies have indicated that polymorphisms in DRD4 and DAT1 are associated with cADHD (Brookes K. et al., 2006; Gornick et al., 2007), while results from a case-control study did not find any association between these two genes and ADHD (Johansson et al., 2008). Moreover, studies attempting to replicate the genetic associations have yielded mixed results. Several studies have suggested that DRD4 is more strongly associated with inattentive symptoms than hyperactive-impulsive symptoms of ADHD (McCracken et al., 2000; Gizer and Waldman, 2012), while other studies showed that DRD4 was implicated in both inattentive and hyperactive symptoms (Lasky-Su et al., 2007; Bidwell et al., 2011). Despite evidence of a strong genetic contribution to ADHD, the inconsistent findings from genetic association studies may result from the relatively small effect sizes, with each gene only accounting for a small proportion of the overall ADHD risk (Gizer et al., 2009).

Increasingly, findings suggest that the genes implicated in cADHD may play different roles in adults with ADHD (Franke et al., 2012). Longitudinal twin studies have found that although many of the same genetic influences operating in childhood continue to explain the total variance in ADHD symptoms at later age periods, their influence on ADHD symptoms declines, or alter, over time (Chang et al., 2013). For example, the DAT1 10/6 haplotype has been associated with ADHD in children, while the 9-repeat and 9/6 haplotype of DAT1 was associated with persistent or adult ADHD (Franke et al., 2008; Brown et al., 2011). Still, studies examining the effects of genes on the persistence of ADHD have produced inconsistent results. Some studies have found that the DRD4 7-repeat (7R) allele was associated with persistent ADHD (Biederman et al., 2009; Langley et al., 2009), while other studies found that the patients with persistent ADHD symptoms were less likely have DRD4 7R allele (Shaw et al., 2007). At the same time, there are also new genes that emerge at a later age which seem to contribute to changes in symptoms (Chang et al., 2013). The differential genetic associations with childhood and adult ADHD suggest that ADHD is a developmentally complicated phenotype characterized by both continuity and alteration of the genetic influences across the life span (Faraone et al., 2006; Biederman et al., 2010; Chang et al., 2013). Intermediate phenotypes were suggested to play a role in mediating the associations between clinical phenotypes and genes in mental disorders (Gottesman and Gould, 2003). The most commonly reported neurocognitive traits linked to ADHD, such as EF, attention, arousal and working memory, may serve as intermediate phenotypes that partially mediate the gene × clinical phenotype interactions in childhood and adult ADHD.

Besides small sample sizes and other sampling factors, etiological heterogeneity is likely to contribute to the inconsistent findings of genetic studies in ADHD. Indeed, such heterogeneity is ubiquitous across psychiatric disorders, with cumulative risk likely conferred by multiple genes of small effect, hence the shift in psychiatric genetics towards establishing links with neurobiological endophenotypes rather than syndromes. Several recent studies found new genetic regions associated with ADHD-related endophenotypes, including Latrophilin 3 gene, Fibroblast growth factor 1 gene, Cholinergic receptor, nicotinic, alpha 4, et cetera (Rommelse et al., 2008; Mastronardi et al., 2016). In addition, genome-wide association study (GWAS) is a novel approach to performing hypothesis-free analyses for searching disease-specific risk genes of considerably smaller effect. The results from GWAS showed that common genetic variants, e.g., CHRNA7, CHMP7, NT5C2, RPL13AP3, link with the phenotypes shared in multiple psychiatric disorders, including schizophrenia, bipolar disorder, anxiety, major depressive disorder and ADHD, while none of these genetic variants are robustly associated with ADHD (Franke et al., 2009; Neale et al., 2010; Stergiakouli et al., 2012; Thapar et al., 2012; Cross-Disorder Group of the Psychiatric Genomics Consortium, 2013; Xu et al., 2017; Brainstorm Consortium et al., 2018).

Environmental risk factors, including maternally related prenatal risks, pregnancy and birth complications, traumatic brain injuries and other external factors, have also been linked to ADHD (Lahey et al., 2009; Thapar and Rutter, 2009; Froehlich et al., 2011a; Thapar et al., 2012; Van Batenburg-Eddes et al., 2013; Adeyemo et al., 2014; Glover, 2014; Silva et al., 2014; Chang et al., 2018; Saez et al., 2018; Schwenke et al., 2018). Prenatal and perinatal factors, including maternal alcohol consumption and smoking (Yoshimasu et al., 2009; Silva et al., 2014; Schwenke et al., 2018), maternal stress (Grizenko et al., 2008; Van Batenburg-Eddes et al., 2013; Glover, 2014), and low birth weight and prematurity (Mick et al., 2002; Thapar et al., 2013) are frequently associated with ADHD. Exposures to other toxins in prenatal and postnatal life have also been considered as increasing the risk of ADHD (Thapar et al., 2013). In particular, organophosphate pesticides, polychlorinated biphenyls, and lead may damage the neural systems implicated in ADHD (Nigg, 2008; Chang et al., 2018). Damage to the brain after birth due to traumatic brain injury has also been considered as a risk factor for ADHD (Pineda et al., 2007; Adeyemo et al., 2014). Multiple indicators of psychosocial adversity, including conflict/parent-child hostility, family adversity and low income, severe early deprivation, have also been found to be associated with ADHD (Pheula et al., 2011). Although there are biologically plausible mechanisms through which these risks could contribute to ADHD, it remains controversial about whether the associations of these environmental risks are directly causal. For example, studies found that children’s ADHD symptoms impact mother-son hostility, rather than the hostility having a causal effect on ADHD (Lifford et al., 2009). However, such statement could not be applied on other environmental risk factors, such as perinatal complications or lead exposure.

Genes and environment do not work independently of each other (Nigg et al., 2010). Studies have explored ways in which the inherited genetic factors might interact with environmental risk factors to influence ADHD development and outcomes. The DAT1 gene has been found to interact with maternal smoking and alcohol use during pregnancy (Brookes K.-J. et al., 2006), while other studies have failed to replicate these results (Becker et al., 2008). Interaction between DAT1 and maternal alcohol use during pregnancy has also been reported to decrease risk for ADHD (Brookes K.-J. et al., 2006). Using a sample of twin pairs, one study found that the interaction between DAT1 9R-allele or DRD4 7R-allele and prenatal smoke exposure increased risk for combined-type ADHD by nine-fold (Neuman et al., 2007). In addition to increasing susceptibility to prenatal adversities, the DAT1 gene has also been found to increase risk for ADHD in the presence of psychosocial adversity. Specifically, the DAT1 10R-6R allele has been found to moderate the effects of early institutional deprivation (Stevens et al., 2009) and psychosocial adversity (Laucht et al., 2007) on ADHD risk. DRD4 has been reported to significantly interact with high stress level during pregnancy and some chemical toxins, e.g., dimethyl phosphate, in children with ADHD (Grizenko et al., 2012; Chang et al., 2018). Nevertheless, these findings have either not been replicated or inconsistent.

Results from existing studies reviewed above suggest that multiple genetic and environmental risk factors with small individual effect sizes contribute to the heterogeneity of ADHD. These etiological risk factors may interact with each other and the complex developmental neural mechanisms, together result in the diverse clinical profiles and outcomes of ADHD.

## Heterogeneity of Clinical Profiles of ADHD

Most diagnoses of ADHD are made in school-aged children. ADHD, according to DSM-5, requires symptoms to present in multiple settings before the age of 12 years. However, the course and outcome of cADHD are highly heterogeneous. Cross-sectional studies have found that ADHD-related symptoms have development-specific features, with motor restlessness, aggressive and disruptive behaviors commonly observed in preschoolers, disorganized, impulsive, and inattentive symptoms more typically presented in adolescents and adults (Wilens et al., 2009). Long-term follow-up studies suggest that hyperactive and impulsive behaviors tend to decrease with age, while inattentive symptoms show greater persistence and may stay life-long (Biederman et al., 2000; Molina et al., 2009). Besides age effects, gender-related differences are also observed in ADHD. Boys are more likely to be diagnosed with ADHD than girls. In the 2011 National Health Interview Survey, the estimated prevalence of ADHD in males was 12 percent; by contrast, in females the estimated prevalence was only 4.7 percent (Perou et al., 2013). Meanwhile, symptom profiles differ between males and females, with females more likely to be diagnosed with predominantly inattentive presentation (Biederman et al., 2005).

Recently, an increasing number of adolescents and young adults have presented to clinics with ADHD symptoms started after 12 or even later in life (Moffitt et al., 2015). A recent longitudinal study found that 2.5%–10.7% of subjects with ADHD first emerge in adolescence or adulthood, with the majority of adults with ADHD (67.5%–90.0%) not experiencing symptoms in childhood (Agnew-Blais et al., 2016). It is still debatable about whether adult ADHD is defined as a childhood-onset neurodevelopmental disorder or not (Moffitt et al., 2015; Agnew-Blais et al., 2016). Results from a recent large sample Multimodal Treatment Study of ADHD (MTA) suggest that common sources of impairing late-onset ADHD symptoms in adolescence and young adulthood were heavy substance use and comorbid psychiatric or learning problems (Sibley et al., 2018).

Although DSM-5-based diagnosis of ADHD offers a common language and standard criteria for identification of the disorder and its sub-types (presentations), emotion lability-based sub-types were also suggested. Deficient emotional self-regulation has been suggested to be a core component of ADHD (Barkley, 2010). A recent meta-analysis of 77 studies with a total of 32,044 participants observed the associations of ADHD with impaired emotional reactivity/negativity/liability, and empathy/callous-unemotional traits of emotional regulation (Graziano and Garcia, 2016). A study conducted by Karalunas et al. (2014) attempted to define three distinct subtpes of ADHD based on temperament profiles, including a “Mild” type, whose members are characterized only by deficits in core ADHD symptom domains; a “Surgent” type, characterized by high levels of positive approach-motivated behaviors and activity level, shorter cardiac pre-ejection period, parasympathetic withdrawal in response to positive emotions, and atypical amygdala connectivity to medial frontal areas; and an “Irritable” type, characterized by high levels of negative emotionality, weak parasympathetic response to negative emotional stimuli, reduced amygdala-insula connectivity, and a doubling of risk for onset of new behavioral or emotional disorders.

Nevertheless, categorical classification system has its shortcomings regarding to what is the best cut-off thresholds of the symptoms characterized as a dimension, the large number of intermediate cases, comorbidities, etc. (Lilienfeld and Treadway, 2016). The NIH Research Domain Criteria (RDoC) represents a new research framework for investigating ADHD by integrating multi-level information (from genomics and circuits to behavior and self-reports) to explore basic dimensions of functioning that span the full range of human behavior from normal to abnormal. The goal of RDoC is to understand the nature of mental health and illness in terms of varying degrees of dysfunctions in general psychological/biological systems (Harkness et al., 2014; Lilienfeld and Treadway, 2016).

Treatment strategies for ADHD symptoms include medication-based, behavior-based, and combined interventions (Antshel et al., 2011; Sibley et al., 2014). Stimulant medications that affect the dopaminergic system, including methylphenidate (Ritalin, Concerta, Metadate, Methylin) and certain amphetamines (Dexedrine, Dextrostat, Adderall), are most commonly prescribed for ADHD. Besides medications, behavior-based treatments, including education and/or behavior therapy, and social skills training have also been implemented in practice for ADHD interventions (Pelham et al., 2016; DuPaul et al., 2017; Anastopoulos et al., 2018; Chacko et al., 2018). Nevertheless, there is yet no curative treatment for ADHD without thoroughly understanding its heterogeneous and developmental pathophysiological mechanisms.

Psychiatric and behavioral comorbidities, such as depression, anxiety disorder, bipolar disorder, substance use, and personality disorders, often co-occur with ADHD and result in increased difficulties for appropriate diagnoses and treatments (Barkley and Brown, 2008; Rosler et al., 2010; Mao and Findling, 2014; Katzman et al., 2017). Although different pharmacological treatment strategies have been applied to ADHD patients with various comorbidities, evidence from a large body of studies showed that treatment responses from different patients are widely different in terms of the types of pharmacological treatments, dosage requirements, tolerability, response rates, and adverse-event profiles (Spencer et al., 1996; Efron et al., 1997; Pliszka, 2007; Newcorn et al., 2008; Victor et al., 2009; Wilens et al., 2011; Hodgkins et al., 2012). Multiple factors may contribute to the treatment response heterogeneity in ADHD. For instance, the treatment response of methylphenidate was suggested to rely on inter-individual variability in the amount of dopamine released by neurons (Volkow et al., 2002; Berridge et al., 2006; Hannestad et al., 2010). Certain polymorphisms, such as the 40-pb variable number tandem repeat polymorphism, noradrenaline, and serotonin transporter genes, were also suggested to be associated with the treatment response to methylphenidate (Winsberg and Comings, 1999; Yang et al., 2004; Thakur et al., 2010; Froehlich et al., 2011b; Bidwell et al., 2017; da Silva et al., 2018). However, recent meta-analyses did not support this polymorphism association hypothesis (Kambeitz et al., 2014; Bonvicini et al., 2016). Treatment response heterogeneity in ADHD, especially those with other psychiatric and behavioral comorbidities, should be more closed investigated in future.

## Heterogeneity of Neurocognitive Impairments and Biological Substrates of ADHD

Heterogeneous neurobehavioral deficits in domains of sustained attention or vigilance, EF, working memory, and self-regulation, have been frequently reported in ADHD, even after controlling the effect of ADHD presentation, age and gender (Nigg et al., 2005; Willcutt et al., 2005). A number of theoretical models of the neurobiological and pathological substrates of ADHD have emerged in the past three decades, aimed at providing systematic guides for more effective strategies in diagnosis and treatment. These models included: (1) cognitive and motivational impairment models (Tannock et al., 1995; Barkley, 1997; Sonuga-Barke, 2003; Martinussen et al., 2005; Martinussen and Tannock, 2006; Rogers et al., 2011; Mawjee et al., 2017; Simone et al., 2018); (2) cognitive-energetic model (CEM; Sergeant, 2000); and (3) neurodevelopmental model (Halperin and Schulz, 2006).

### Cognitive and Motivational Impairment Models

Traditional explanations of ADHD pathology have been based on impaired signaling of delayed rewards arising from disturbance in motivational processes (Sonuga-Barke, 2003), and neurocognitive dysfunctions, including abnormal working memory (Tannock et al., 1995; Martinussen et al., 2005; Rapport et al., 2008; Mawjee et al., 2017; Simone et al., 2018) and executive dysfunction due to deficient inhibitory control (Barkley, 1997). Several investigators have suggested that working memory impairment is a core neurocognitive deficit of ADHD (Tannock et al., 1995; Martinussen et al., 2005; Rapport et al., 2008; Mawjee et al., 2017; Simone et al., 2018). Working memory processing allows for temporary storage, maintenance, and manipulation of sensory and cognitive information, and provides an interface between perception, attention, memory, and action, and is crucial for higher order cognitive processes (Baddeley, 1992). As such, it is responsible for recognition of externally presented stimuli. Working memory impairment has been linked to inattentiveness in ADHD (Lui and Tannock, 2007; Rogers et al., 2011). Previous studies indicate that deficits in working memory, including verbal and visuospatial short-term memory and verbal central executive memory, contribute to inattention symptoms (Willcutt et al., 2005). Working memory task-based functional magnetic resonance imaging (fMRI) and positron emission tomography (PET) studies demonstrated that both children and adults with ADHD, predominantly inattentive and combined presentations, had reduced brain activities in working memory processing related brain regions, including prefrontal cortex (PFC) and caudate (Fassbender et al., 2011; Roman-Urrestarazu et al., 2016). A resting state function connectivity study found that abnormal thalamo-caudate connectivity was associated with impaired spatial working memory in patients with ADHD patients (Mills et al., 2012).

Barkley’s self-regulation model (Barkley, 1997) attempted to explain the symptoms of hyperactivity and impulsivity, by linking impaired inhibitory processing with executive dysfunction in four domains: (1) nonverbal working memory; (2) internalization of speech (verbal working memory); (3) self-regulation of affect-motivation-arousal; and (4) reconstitution (planning and generativity). By definition, behavioral inhibition is the suppression of an immediate response that creates a time lag to allow for subsequent EFs. Barkley et al. (2001) suggested that individuals with ADHD suffer a primary deficit in behavioral inhibition, which leads to secondary impairments in EFs and motor output. A number of existing behavioral studies have demonstrated poor response inhibition capacity in patients with ADHD when performing Stop-Signal and Go/No-go tasks (Desman et al., 2008). Abnormal functional activation and connectivity in OFC and DLPFC have been suggested to influence behavioral inhibition, reward-motivation issues, working memory, attention, planning, and executive control in children with ADHD (Schulz et al., 2004; Bush, 2010; Wilbertz et al., 2012; Janssen et al., 2015; Zhang et al., 2017; Tsvetanov et al., 2018). One prevailing theory regarding the neurobiological basis of ADHD suggests the fronto-striatal network as a probable substrate of cognitive and behavioral impairments seen in ADHD (Bush et al., 2005; van Ewijk et al., 2012). A resting-state fMRI study supported this theory by demonstrating the linkage of stronger fronto-striatal functional connectivity and better EF, especially response inhibition, in patients with ADHD (Li et al., 2014). Moreover, existing studies also observed the involvement of the fronto-striatal circuit in working memory impairment and delay aversion in ADHD (Castellanos et al., 2006; Vaidya and Stollstorff, 2008; Darki and Klingberg, 2015; Bollmann et al., 2017). Nevertheless, this model may only support the role of deficient behavioral inhibition as a central feature of ADHD, the mechanisms of how the primary deficits of inhibitory control is associated with inattentive deficits is not explained. Furthermore, a meta-analysis conducted by Willcutt et al. (2005) including 83 studies concluded that although ADHD is associated with weakness in response inhibition, working memory, vigilance and planning, such EF deficits were neither necessary nor sufficient for the presence of ADHD.

### Cognitive-Energetic Model

The CEM proposes that the overall efficiency of information processing is determined by the interplay of three levels: (1) cognitive processes, including encoding, central processing and response organization; (2) energetic pools, including arousal, activation and effort; and (3) management/EF, which encompasses both top-down and bottom-up processes and draws attention to the fact that ADHD is linked to defects at these three levels (Sergeant, 2000). According to this model, effective cognitive functioning relies on an optimal energetic state wherein arousal and activation are optimally adjusted to actual task demands (Sergeant, 2005). When the adjustment is suboptimal, i.e., when under- or over-activation occurs, task performance deficits are predicted to occur. Several studies have directly tested this performance pattern hypothesis and found their results to be supportive to the model predictions (Wiersema et al., 2006; Metin et al., 2012).

Thus, the CEM describes suboptimal performance in ADHD as a response modulation deficit that is triggered by contextual factors such as event rate. It explained a phenomenon that performance can be improved to normal levels under conditions that are thought to increase arousal and/or motivation, such as the provision of external rewards (Konrad et al., 2000; Slusarek et al., 2001) or the faster presentation of stimuli (Scheres et al., 2001). The model suggested that although many mental disorders have common deficiencies in EF domains, ADHD may be distinguishable either at an energy level or at specific elementary cognitive stages, such as arousal and motor activation (VaezMousavi et al., 2007).

### Neurodevelopmental Model

In the past decade, the neural mechanisms that determine the diverse developmental trajectories of ADHD have been increasingly investigated. Halperin and Schulz (2006) proposed a double dissociation model in which the early onset of cADHD is associated with dysfunctional subcortical structures, including thalamus and brainstem, that remain static throughout the lifetime; while diminution of symptoms over development is associated with optimal development of PFC (Halperin and Schulz, 2006). A number of existing cross-sectional and longitudinal studies in patients with cADHD support the role of subcortical structures in the emergence of ADHD by showing that reduction of GM volume in thalamus and striatum, and disruption of WM integrity in the major association and projection tracts, contribute to the etiology of ADHD (Xia et al., 2012; Cortese et al., 2013; Shaw et al., 2015). However, decreased frontal lobe, cerebellar and striatal volumes, delayed posterior to anterior cortical maturation, and dysfunction of fronto-striatal and thalamo-cortical networks were also demonstrated to involve in the emergence of ADHD (Bush et al., 2005; Li et al., 2012; Xia et al., 2012; Shaw et al., 2013). The role of cortical maturation in the developmental diminution of ADHD symptoms has also received mixed support. A number of neuroimaging studies have shown that reduced symptoms over development correlate with the maturation of PFC and related circuitry (Mackie et al., 2007; Makris et al., 2007; Clerkin et al., 2013; Shaw et al., 2013; Francx et al., 2015) and EFs subserved by these circuits (Halperin et al., 2008; Rajendran et al., 2013; Szekely et al., 2017). However, these studies also reported that ADHD symptom remission also link to normalized brain activities in anterior cingulate cortex and cerebellar regions (Francx et al., 2015; Szekely et al., 2017).

In summary, this section reviewed three theoretical models of ADHD; cognitive and motivational impairment models, the CEM, and the neurodevelopmental model. Each of these models partially explains the symptomology of ADHD. In particular, the working memory and self-regulation models may describe different components of ADHD, with the working memory model more linked with the inattentive symptoms and the self-regulation model more linked to impaired inhibitory control and hyperactive/impulsive presentation. The CEM tried to explain the disorder in three distinct levels, including deficits in cognitive processes, energetic pools, and EF. While the neurodevelopmental model proposed a double dissociation model that the early onset of cADHD is associated with non-cortical dysfunction, including thalamus and brainstem, that remains static throughout the lifetime; and diminution of symptoms over development was associated with optimal development of PFC. Findings from multiple studies partially support these models. However, these existing studies have focused on individual hypotheses, without addressing the potential interactions of these theoretical models (Dias et al., 2013).

## Discussion

Existing data clearly indicate that ADHD, as defined by the key behavior features of inattention and/or hyperactivity/impulsivity, is a highly complex and heterogeneous disorder in terms of its multi-factorial etiological risk factors, diverse neurocognitive impairments and comorbid problems. There is yet no curative treatment for ADHD. The most commonly prescribed medications for treating ADHD symptoms include methylphenidate, amphetamines, and non-stimulant drugs. In addition to medications, behavior therapy, social skills training, exercise, and nutrition management have also been implemented in practice for interventions of ADHD. Treatment responses from patients have been found to vary widely in terms of the types of optimal pharmacological treatments, dosage requirements, tolerability, response rates, and adverse-event profiles. Multiple factors, such as psychiatric and behavioral comorbidities, symptom severity levels, and inter-individual variability in the amount of dopamine released by neurons, may contribute to the treatment response heterogeneity in ADHD. Further investigations are to better understand the treatment response heterogeneity in ADHD.

Heterogeneous neurobehavioral deficits in cognitive domains, such as sustained attention or vigilance, EF, working memory, and self-regulation, have been frequently reported in ADHD (Nigg et al., 2005; Willcutt et al., 2005). Several theoretical models have attempted to link the clinical profiles of ADHD with the core neurocognitive impairments, the degrees of cognitive-energetic interactions, and the neurodevelopment-related variation. Findings from multiple studies partially support these models. However, these existing studies have focused on individual hypotheses, without addressing the potential interactions of these theoretical models (Dias et al., 2013). In addition, none of these theoretical models completely address the heterogeneity of ADHD.

Nevertheless, none of the existing clinical or translational research findings have been translated into a meaning that can directly guide treatment and intervention for ADHD. Herein, it is time for future research to allocate greater resources to understanding biologically more homogeneous subgroups of ADHD, that hold the potential to facilitate the development of more tailored intervention strategies in ADHD. As reviewed above, familial history has been found to be the most significant risk factor for the emergence and persistence of ADHD (Chang et al., 2013; Bonvicini et al., 2018; Uchida et al., 2018). Clinical studies suggest that heritability significantly contributes to impaired EF in ADHD (Bidwell et al., 2007; Friedman et al., 2008). Neuroimaging and neuropsychological studies in siblings found that compared to controls, patients with ADHD and their unaffected siblings had smaller inferior, medial and orbitofrontal GM volume (Pironti et al., 2014; Bralten et al., 2016), reduced superior frontal activations and fronto-striatal functional connectivity during performance of go/nogo tasks, correlated with lower WM integrity in prefrontal lobe (Rapoport and Shaw, 2008; Godinez et al., 2015), as well as significant cognitive deficits in EF (Friedman et al., 2008; Godinez et al., 2015). All these studies suggest that some frequently observed brain and behavioral abnormalities associated with frontal lobe and EF in ADHD may have heritable patterns. These findings suggest that familial ADHD may represent a biologically more homogeneous subgroup. Examining neural substrates of familial vs. non-familial ADHD could facilitate the development of more tailored interventions that target the distinct underlying neural anomalies characteristic of these more homogeneous subgroups. This, in turn, could lead to novel treatments for which positive family history of ADHD could serve as an easily ascertained moderator of treatment response in children. In addition, subgroups sorted by other factors, such as environment risk factors, clinical profiles, neurocognitive impairments, should be closely investigated. For example, research on subgroups sorted by environmental factors, such as prenatal substance exposure, heavy metal and chemical exposure, or nutritional factors, may lead us to a new insight into these etiological more homogeneous subgroups of the disorder.

We also discussed possible moderators contributing to the inconsistency of the existing studies in ADHD. First of all, the sample biases may directly impact the consistency of the studies. Limited sample size, clinical heterogeneity in terms of comorbidities, gender effects, diverse courses and outcomes, varying etiological sources, also play their roles (Hyman, 2007; Kapur et al., 2012; Karalunas et al., 2014). Nevertheless, the current categorical classification framework has limitations in dealing with the inconsistency of research on such a highly heterogeneous disorder. The NIH RDoC may provide an improved framework that allows us to explore impairments in each basic dimension of the cognitive functioning by integrating multi-level information from the etiological factors, neural mechanisms, and behavioral deficits.

## Author Contributions

XL and JH designed the study. YL and DW conducted literature search and wrote the initial draft. All the authors contributed to manuscript writing and revisions.

## Conflict of Interest Statement

The authors declare that the research was conducted in the absence of any commercial or financial relationships that could be construed as a potential conflict of interest.
